# Serial dependence in facial identity perception and visual working memory

**DOI:** 10.3758/s13414-023-02799-x

**Published:** 2023-10-04

**Authors:** Anette Lidström

**Affiliations:** Department of Psychology, Lund University, Allhelgona kyrkogata 16A, 223 50 Lund, Sweden

**Keywords:** Serial dependence, Face perception, Task relevance, Temporal dynamics, Visual working memory

## Abstract

Serial dependence (SD) refers to the effect in which a person’s current perceptual judgment is attracted toward recent stimulus history. Perceptual and memory processes, as well as response and decisional biases, are thought to contribute to SD effects. The current study examined the processing stages of SD facial identity effects in the context of task-related decision processes and how such effects may differ from visual working memory (VWM) interactions. In two experiments, participants were shown a series of two sequentially presented face images. In Experiment 1, the two faces were separated by an interstimulus interval (ISI) of 1, 3, 6, or 10 s, and participants were instructed to reproduce the second face after a varying response delay of 0, 1, 3, 6, or 10 s. Results showed that SD effects occurred most consistently at ISI of 1 s and response delays of 1 and 6 s consistent with early and late stages of processing. In Experiment 2, the ISI was held constant at 1 s, and to separate SD from VWM interactions participants were post-cued to reproduce either the first or the second face. When the second face was the target, SD effects again occurred at response delays of 1 and 6 s, but not when the first face was the target. Together, the results demonstrates that SD facial identity effects occur independently of task-related processes in a distinct temporal fashion and suggest that SD and VWM interactions may rely on separate underlying mechanisms.

Using past visual input to interpret what we currently see makes the external world more predictable and manageable. Serial dependence (SD) refers to the effect in which a person’s current perceptual judgment is attracted toward recent stimulus history (Fischer & Whitney, 2014). The effect typically manifests as a systematic bias in responses to the target stimulus of the current trial toward the target stimulus of the previous trial and occurs as a function of how close the stimuli are in time, space, and similarity (Kiyonaga et al., 2017). SD has been found for a variety of objects and features, such as orientation (Cicchini et al., 2018; Fischer & Whitney, 2014; Fritsche & de Lange, 2019; van Bergen & Jehee, 2019), visual search (Manassi et al., 2019), motion direction (Bae & Luck, 2020), and facial attributes such as identity (Hsu & Lee, 2016; Liberman et al., 2014; Turbett et al., 2019), emotional expression (Liberman et al., 2018; Mei et al., 2019), and attractiveness (Pegors et al., 2015; Xia et al., 2016). It is proposed that SD is a universal mechanism that promotes visual stability and continuity by integrating visual input over short periods of time (Burr & Cicchini, 2014; Fischer & Whitney, 2014). Although perceptual and memory processes, as well as response and decisional biases are thought to contribute to SD effects (Whitney et al., 2022), the functional loci of such effects are not well understood. In particular, the question of how important postperceptual decision, and memory processes are for the manifestation of SD needs further clarification. Here, two novel experiments are reported examining the time course of SD for facial identity in the context of task-related decision processes.

Task-related information is generally prioritized for processing over task-irrelevant information (Gazzaley, 2011; Schmidt et al., 2002). Recent evidence suggests that SD occurs only when a prior stimulus is relevant to a task and is intentionally subjected to memory or decision-making processing (Bae & Luck, 2020; Pascucci et al., 2019). For example, Bae and Luck (2020) used an experimental task in which participants were instructed to attend to two-dimensional motion direction and color stimuli, and included a postcued response task in which only one of the two dimensions was reported. Results showed that SD manifested reliably only from the previously reported dimension and that mere attention to the unreported dimension was not sufficient to manifest SD. Likewise, when participants are explicitly instructed to only attend to the orientation of a grating on the previous trial without responding to it, responses in the current trial are repelled (i.e., pulled away from) by the target on the previous trial (Pascucci et al., 2019). Moreover, when judgments are made about one of two successive stimuli presented in the same trial: Responses are repelled by the task-irrelevant stimulus within a trial, while simultaneously being attracted toward the task-relevant stimulus of the previous trial (Czoschke et al., 2019; Pascucci et al., 2019; Pascucci & Plomp, 2021).

Task-relevant information is thought to be encoded more efficiently during visual working memory (VWM) maintenance (Jackson et al., 2017; Serences et al., 2009) and in addition to decision processes, SD is thought to arise from post-perceptual memory processes. Bliss et al. (2017) found stronger SD effects at longer compared with shorter intertrial intervals and delays between stimulus and response (peaking at 3 and 6 s, respectively) for spatial position judgments, suggesting that the effect is due to VWM processes. A repulsive effect was found at a delay of 0 s between target and response, which led the authors to conclude that the representation formed in early perception may be immune to interference from the stimulus in the previous trial. The results of Bliss et al. are consistent with those for orientation judgments, where SD has also been interpreted as a postperceptual effect arising from memory traces of task-relevant target stimuli in the previous trial (Fritsche et al., 2017). Taken together, this suggests that SD depends on whether a prior stimulus is explicitly used in the decision process or intentionally memorized with respect to a particular task.

In addition to task-relevance, a stimulus is processed by its emotional valence and behavioral significance, which determine its relevance and influence how efficiently the stimulus is processed (Compton, 2003; Kauschke et al., 2019). Faces are behaviorally relevant stimuli that are thought to be automatically processed due to a close interaction between face perception and attention (Compton, 2003; Devue et al., 2012; Palermo & Rhodes, 2007; Sato & Kawahara, 2014). For example, faces that are irrelevant to a task and presented in rapid succession have been found to interfere with the identification of a subsequent face (Ariga & Arihara, 2017; Eitam et al., 2014), and facial identity information has been found to be retained in VWM despite being task irrelevant (Schweinberger et al., 2004). Moreover, task-irrelevant faces presented during a retention interval have been found to influence recall of a previously memorized target face (Mallett et al., 2020).

While previous studies regarding post-perceptual processes in SD effects have largely focused on simple stimuli, there is a small but growing body of research using facial stimuli (Liberman et al., 2014; Mei et al., 2019; Pegors et al., 2015). In one experiment, Mei et al. (2019) showed participants two sequentially presented images of facial expressions in each trial drawn from a morphed continuum of facial expressions ranging from happy to sad. The first stimulus on each trial looked either happy or sad, and participants responded by judging which of the two facial expressions they perceived as happier. Results showed that on trials with longer interstimulus intervals (2,500-ms mask + 250-ms fixation), responses were pulled toward the preceding facial expression. However, SD effects were not reproduced for shorter interstimulus intervals (50-ms mask + 250-ms fixation), suggesting that the effect depended on how long the representations were held in VWM.

While emotional expression is a changeable attribute that requires continuous updating, SD also appears to arise as a result of postperceptual decision processes for stable facial attributes such as attractiveness. For example, Pegors et al. (2015) showed that attractiveness judgments are pulled toward the prior response decision, while simultaneously repelled by the average attractiveness of prior faces (but see Xia et al., 2016). However, attractiveness judgments rely on mechanisms beyond visual information, such as mate selection, gender, and biological factors such as the observer’s hormone levels (Kou et al., 2020). The combination of these factors may therefore contribute to SD for attractiveness at other processing levels rather than on the basis of visual stimulus information.

Facial identity is also a stable attribute that, unlike attractiveness, is processed through a complex combination of multidimensional visual cues such as shape, texture, and color (Bruce & Young, 1986; O'Toole et al., 1999; Valentine et al., 2016), and it is not clear whether SD for facial identity occurs independently of post-perceptual processes. Liberman et al. (2014) attempted to rule out that SD for facial identity is a bias due to decision processes by including randomly nested “surprise” no-response trials in the experimental task. On each trial, participants were presented with a face image drawn from a continuum of morphed images, which they reproduced in a continuous adjustment task after a delay of 1,250 ms (1,000-ms mask + 250-ms fixation). The response task was randomly excluded on 50% of all trials. The results showed that SD occurred even when no response was made in the preceding trial, but this does not rule out the possibility that the effect could have arisen from response preparation, because the experimental design contained an element of surprise.

While it is difficult to isolate perceptual and VWM effects from those resulting from response and decisional biases in SD face effects between trials, there is evidence that SD may also occur when two face images are presented within the same trial. For example, Turbett et al. (2019) investigated the relationship between SD and face recognition skills. Here, participants were first trained to recognize two original male and female identities. On each trial, two faces from a morphed continuum between either the original male and female faces were presented in succession, and participants had to identify the second face that appeared. The two presented faces could differ in one of four morph difference magnitudes:±24%, or±12%. To examine SD, a four-alternative forced-choice procedure was used in which participants were asked to identify the second face as one of the original faces in the continuum by pressing a key. Results showed that the second face was more likely to be perceived as the target identity if it was preceded by a face more similar to that identity. Although such within-trial effects might suggest that SD for facial identity can occur independently of postperceptual processes, it was not the aim of Turbett et al. to investigate this, and it was therefore not discussed. In addition, SD is reported to be temporally tuned to a ⁓ 10 to 15-second window back in time and stimuli from the previous trial (Liberman et al., 2014) or the previous motor response (Wagenaar, 1968) may have biased any within-trial effects.

The aim of the present study was to investigate further the processing stages of SD facial identity effects. More specifically, whether encoding a task-irrelevant face is sufficient for SD to manifest and the temporal dynamics of such effects. In two experiments, two successive facial identities were presented on each trial. In Experiment 1, the firstly presented face was always irrelevant to the task, whereas in Experiment 2, postcues were used to manipulate target priority, which also fulfilled the goal of isolating SD from VWM interactions. By manipulating the interstimulus interval between the two faces in Experiment 1, and the delay between the second face and the response in both experiments, the present study uncover the temporal dynamics of SD face effects from early perceptual to VWM stages. A preview of the results shows that SD manifested in a distinct temporal fashion regardless of whether a previous face was merely perceived or intentionally memorized.

## Experiment 1

Building on the work of Liberman et al. (2014), an experimental task was used in which not one, but two successive facial identities were presented on each trial. Participants were explicitly informed that the first face on each trial was irrelevant to the task and should only be attended to. If task-relevant information is prioritized over task-irrelevant information (Gazzaley, 2011; Schmidt et al., 2002), a face that is irrelevant to the task may not be sufficiently processed for SD to manifest. However, there are several lines of evidence that faces are processed more efficiently due to their priority access to attention (Compton, 2003; Devue et al., 2012; Palermo & Rhodes, 2007; Sato & Kawahara, 2014), so SD may still occur from a face that is not relevant to the current task. Moreover, faces may be unintentionally retained in VWM despite being task-irrelevant (Eitam et al., 2014; Schweinberger et al., 2004). Following previous work (Bliss et al., 2017; Mei et al., 2019), the interstimulus interval and delay between the target face and the response were varied in an attempt to shed new light on the relative time course of the processes underlying SD effects in facial identity judgments.

### Method

#### Participants

Twenty-one Psychology students at Lund University participated in Experiment 1. One participant withdrew from the study, leaving a final sample of 20 participants (17 women and three men; age range: 20–50 years). All participants reported normal or corrected-to-normal visual acuity, and all received a gift voucher worth 100 SEK upon completion of each experimental session.

The experiment was conducted in accordance with the guidelines of the Swedish Research Council’s ethics committee and the European Research Council’s ethics committees for research involving human participants. All participants were informed of the experimental procedure, their right to withdraw from the study at any time without consequence, and all participants provided signed consent before taking part in the study.

#### Material

**Fig. 1:**
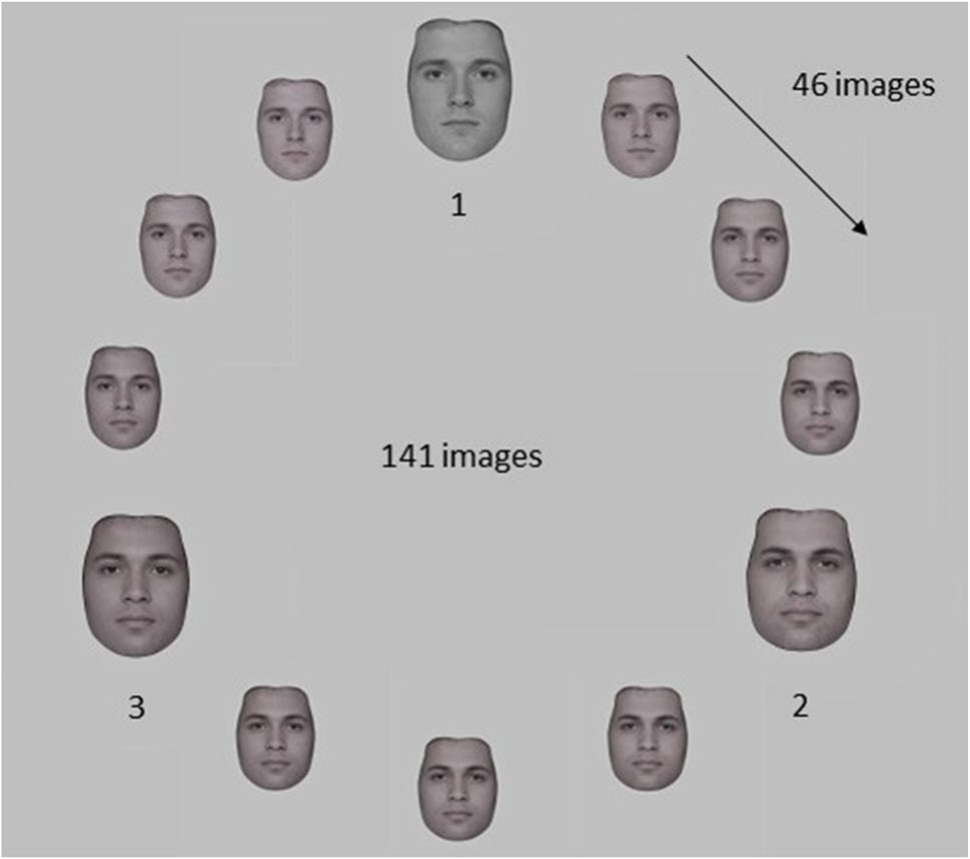
Morphed image continuum. Illustration of the morph wheel. A morph wheel was created by generating 46 morphed images between each pair of three original male face images (141 images in total). The original faces are labelled 1, 2, and 3, and shown at a larger size than the morphed faces. All face images were the same size in the experiment

Stimuli were drawn from a grayscale morphed image continuum derived from three original male faces selected from the Face Research Lab London Set (Debruine & Jones, 2017). As shown in Fig. 1, a set of 46 morphed face images was created between each of the three original face images resulting in a morph wheel of 141 images in total. All faces displayed neutral expressions and were cropped with an oval mask to exclude hairlines and other external features. The morph wheel was created using Webmorph (Debruine, 2018).

All stimuli were presented in the center of a 21-inch ViewSonic G220*f* (40.5 cm × 30.5 cm) monitor (ViewSonic Corporation, Walnut, California) against a light-gray background with a pixel resolution of 1,024 × 768 and a refresh rate of 85 Hz.

#### Procedure

**Fig. 2:**
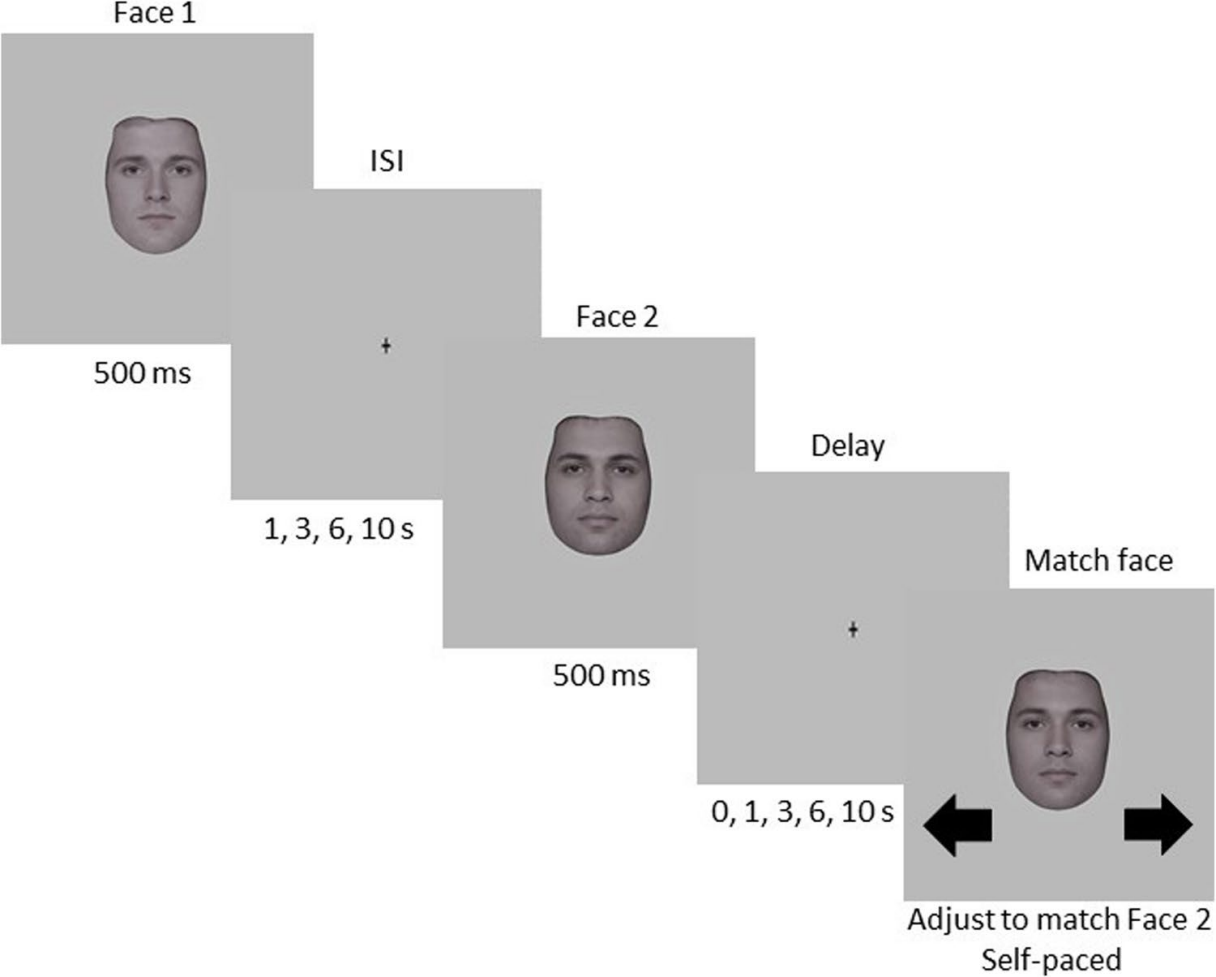
One trial sequence in Experiment 1. Illustration of one trial sequence in Experiment 1. Each trial began with two randomly presented face images (Face 1 and Face 2), each presented for 500 ms and separated by an interstimulus interval (ISI). The ISI varied pseudorandomly for 1, 3, 6, or 10 s, and a fixation cross was present during each ISI. Following Face 2 and before the response, a fixation cross was displayed during a response delay that varied pseudorandomly for 0, 1, 3, 6, or 10 s. Then, a test screen that contained a random adjustment face from the morphed continuum was presented and participants used the left and right arrow keys to match this face to Face 2. The response task was self-paced, and responses were registered by pressing the down arrow key

All participants were tested individually in a dimly lit room, seated at a viewing distance of 57 centimeters from the monitor such that a spatial extent of 1 cm on the monitor corresponded to about 1 degree of visual angle. Each trial comprised two sequentially presented face images (Face 1 and Face 2) randomly drawn from the morph wheel. Figure 2 shows an illustration of an experimental trial. Each face image was presented for 500 ms, separated by an interstimulus interval (ISI) of 1, 3, 6, or 10 s selected pseudorandomly to ensure that each ISI was presented an equal number of times during each experimental session. A fixation cross was present throughout each ISI. Participants were instructed to attend to both Face 1 and Face 2 but informed that they only should respond to Face 2.

Following Face 2 and prior to the response, a fixation cross was present during a response delay of 0, 1, 3, 6, or 10 s (Bliss et al., 2017). Pseudorandom selection ensured that each response delay was presented an equal number of times in each experimental session. Participants then saw a test screen that contained an adjustment face randomly drawn from the morph wheel, which they adjusted to match Face 2 by using the left and right arrow keys (so called, continuous adjust to match response). After selecting a match face, participants pressed the down arrow key to register their response. The adjustment task was self-paced; participants were given as much time as necessary to respond. Following the response, a fixation cross was displayed during an intertrial interval (ITI) of 1 s before the next trial began. Each experimental session comprised 180 trials, and participants took an average of 60 min to complete each experimental session. Each participant completed between 180 and 540 trials in one to three separate sessions, totaling 9,000 trials across all participants.

Power analysis was not considered relevant under the circumstances, as data were pooled across all participants to facilitate the chosen modelling approach, whereby confidence intervals were subsequently determined by resampling methods.

The experimental task was run using MATLAB (The MathWorks, Natick, MA) along with the Psychophysics Toolbox (Version 3) extension (Brainard, 1997; Kleiner et al., 2007; Pelli, 1997) on a Dell Precision 3460 computer (Dell Inc., Round Rock, TX). Timing tests of the experimental setup conducted using the Black Box Toolkit (Plant & Hammond, 2002) verified the consistency of the timings requested by the experiment script.

#### Data analyses

##### Within-trial serial dependence

Trials were first aggregated into twenty different conditions grouped by ISI (1, 3, 6, 10 s) and response delay (0, 1, 3, 6, 10 s). Following Liberman et al. (2014), trials with response times >15 s and adjustment errors exceeding±60 morph steps were defined as random errors or lapses in attention and discarded from further analysis. This resulted in the removal of 4.1% of trials. Adjustment errors were calculated as the shortest, clockwise (+) or counterclockwise (−), distance in morph steps between Face 2 and the response. The shortest distance in morph steps corresponds to the number of morphed images between two faces in the morph wheel. Adjustment errors were then plotted against the shortest, clockwise (+) or counterclockwise (−), difference in morph steps between Face 1 and Face 2, pooled across all participants, by each ISI and response delay condition. In this regard, SD is said to be manifest if responses to Face 2 are pulled in the direction of Face 1 (Fischer & Whitney, 2014).

**Fig. 3:**
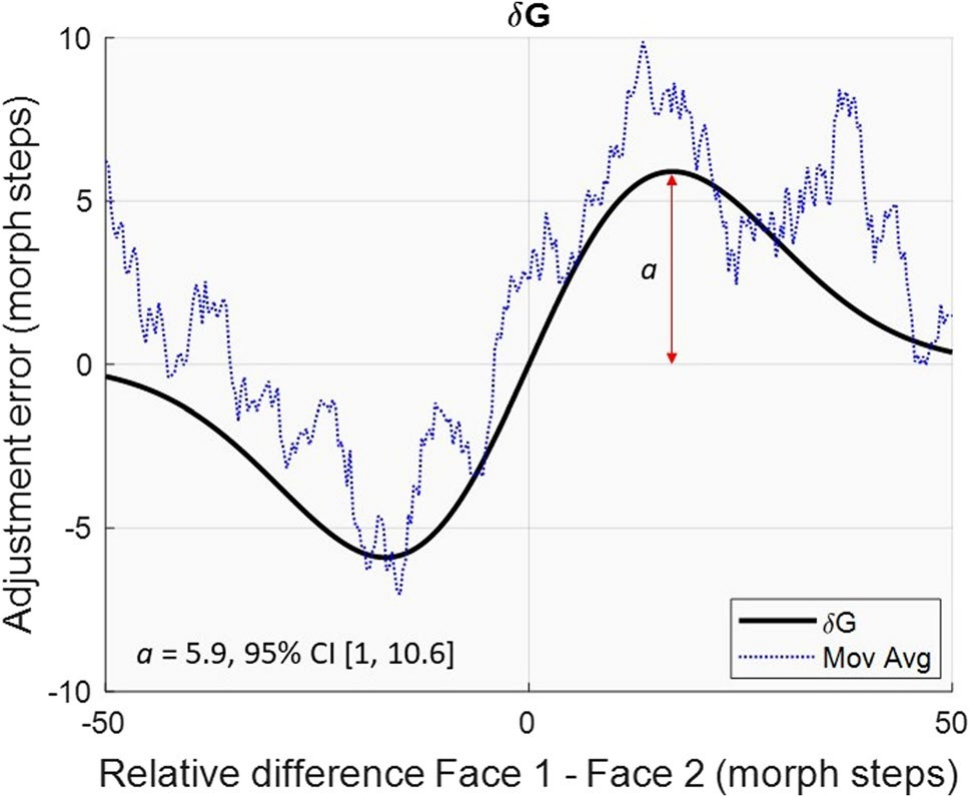
*δG* fit for the 1 s ISI and 6 s response delay condition. Serial dependence for the 1-s ISI and 6-s response delay. Adjustment error (ordinate) as a function of the shortest difference in morph steps between Face 1 and Face 2 (abscissa). The black solid line shows the fit of a *δG* model to the group data and the blue dotted line represents the moving average. Positive values represent clockwise differences. The half-amplitude (*a*) captures the magnitude of serial dependence. (Color figure online)

As a first estimate of SD, a derivative of Gaussian (*δG*) function defined as: *y = f(x)µσxalpha* was fitted to the data averaged across all participants by each ISI and response delay condition, using constrained nonlinear minimization of the residual sum of squares (Johansson, 2020). The parameters in the model are as follows: *f(x)* represents a normal probability density function with mean *µ* and standard deviation *σ*;* y* represents each trial’s adjustment error and *x* is the difference in morph steps between Face 1 and Face 2; *alpha* is a parameter that, when multiplied by *f(x)x*, the first derivative of the Gaussian function, affects the height of the curve. The *σ* parameter controls the width of the curve. The half-amplitude (the highest point in the curve from peak to zero) was used as a measurement of SD magnitude where positive values were interpreted as attractive effects and negative values as repulsive effects. Figure 3 shows the *δG* function fitted to the 1-s ISI and 6-s response delay condition, along with the half-amplitude *a* as an estimate of SD magnitude.

Averaged across all participants and conditions mean adjustment time was 5.4 s, with a standard deviation of 3 s. No statistically significant differences were found between mean adjustment times for the different experimental conditions, all *p*s > .05.

To test for statistically significant SD effects, bootstrapped 95 % confidence intervals (CIs) were computed for each ISI and response delay condition by randomly resampling the data with replacement for 10,000 iterations. A *δG* function was fitted to each resampled dataset resulting in a distribution of 10,000 half-amplitudes defining the boundaries for the 95% CIs.

##### Across-trial serial dependence

SD is by convention measured across trials (i.e., responses to a stimulus in the current trial are pulled toward the previous trial’s target stimulus) and is reported to be temporally tuned to a ⁓10 to 15 s window back in time (Liberman et al., 2014). In the present experiment, it was therefore relevant to determine whether the previous trial’s stimuli or the previous trial’s response influenced responses to Face 2 in the current trial. To this end, three separate *δG* functions were fitted to the data pooled across all participants by each ISI and response delay condition following the same procedures as described above under the header “Within-trial Serial Dependence.” In the first model, the *x* parameter was set to represent the difference in morph steps between Face 2 in the current and previous trial. In the second model, the *x* parameter was set to represent the difference in morph steps between Face 2 in the current trial and Face 1 in the previous trial. In the third model, the *x* parameter was set to represent the difference in morph steps between Face 2 in the current trial and the response in the previous trial. To test for statistically significant half-amplitudes, 95% bootstrapped CIs for each experimental condition were computed following the same resampling procedure as previously described.

##### Nonparametric analysis

**Fig. 4:**
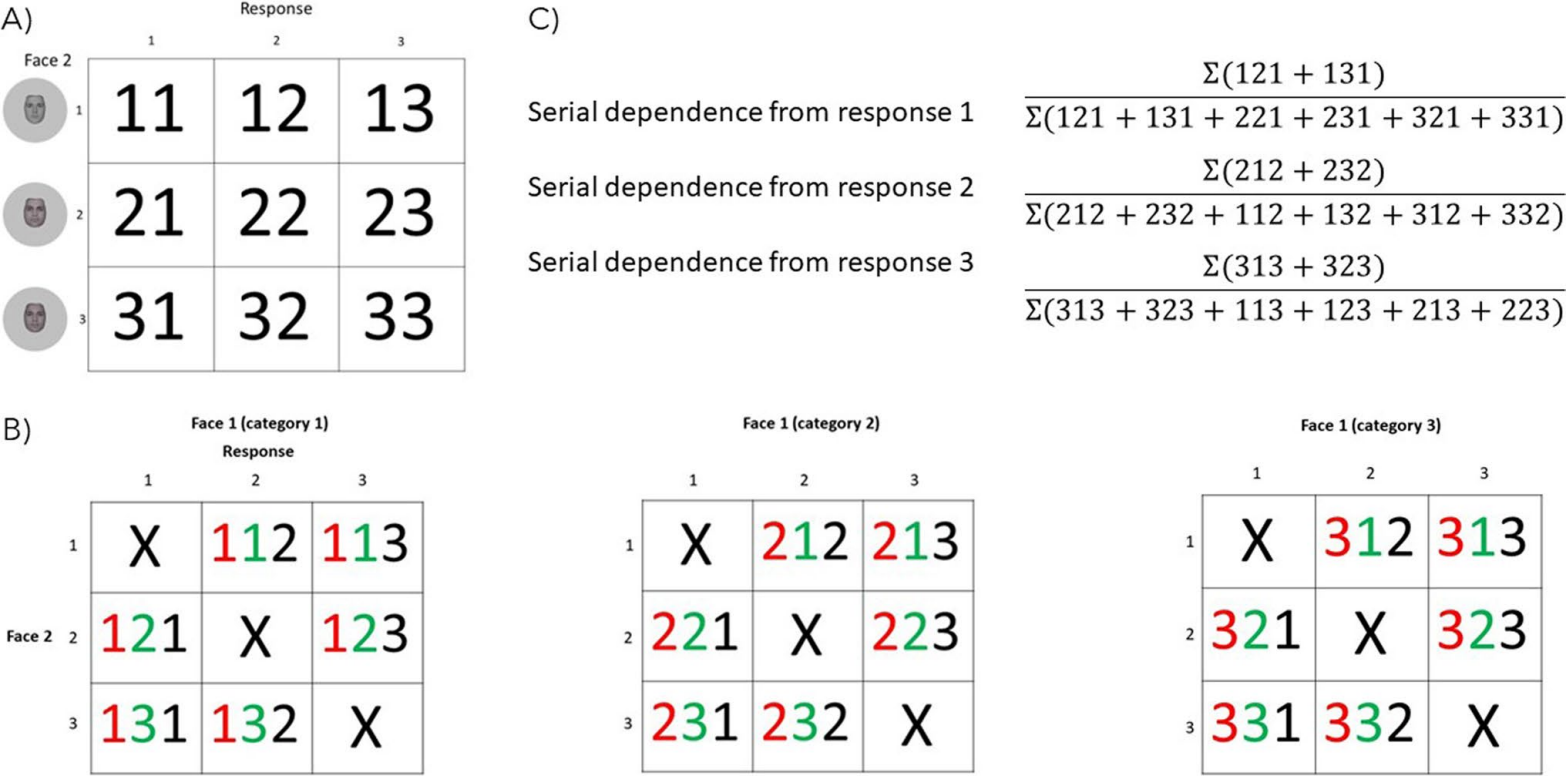
Nonparametric analysis based on categorical classification errors. Categorical measurement of serial dependence. The three original face images from the morphed continuum were set as prototypes for each category (1 / 2 / 3) and category boundaries were set to±23 morph steps from each category prototype. **A** Response frequencies were separated into a matrix depending on the category of Face 2 for each ISI and response delay condition. **B** Classification errors were then separated into three matrixes depending on the category of Face 1. Red = Face 1 category, green = Face 2 category, black = response category. Correct classifications (diagonal) were excluded and only classification errors were considered. **C** Classification errors were summed for all response categories when responses and Face 1 belonged to the same category and then divided by the sum of all classification errors. (Color figure online)

SD is by convention modelled using a *δG* function. Artefactual results, such as a failure to capture SD for larger stimulus differences, can however result as a function of model fitting (see Bliss et al., 2017, for discussion). So, as an additional step, nonparametric analysis of the data based on categorical classification errors was conducted following the procedure of Manassi et al. (2019). First, the morph wheel was divided into three categories according to the three original faces. Each original face (labelled 1 / 2 / 3 in Fig. 1) was set as a prototype for one category and category boundaries were set to±23 morph steps from each of the three original faces. The percentage of classification errors when the adjustment response fell within the prototypical boundaries of Face 1 were then computed and subsequently used as a categorical measurement of SD. A graphical illustration of this nonparametric procedure is shown in Fig. 4.

Responses across all participants by each ISI and response delay condition were first divided into categories (1 / 2 / 3) and the number of classification errors were computed given the category of Face 1. When the category of Face 1 and the classification errors were the same, classification errors for each category were separately summed and then divided by the sum of classification errors for all Face 1 categories. This resulted in a percentage ratio for each classification error category. These percentage ratios were then averaged across the three classification error categories and the chance baseline of 33.33% was subtracted. The resulting index was used as a measurement of how much responses to Face 2 were pulled in the direction of the category of Face 1. Positive indexes were interpreted as attractive effects (i.e., SD) and negative indexes as repulsive effects.

Percentage ratios for each experimental condition across all participants were then bootstrapped for 10,000 iterations and the mean bootstrapped percentage interpreted as a measurement of SD. To test for statistically significant percentage indexes, 95% CIs were computed using the MATLAB *bootci* function. Lastly, correspondence between the category of Face 2 and response category was computed for each experimental condition across all participants as a measurement of classification accuracy. All statistical analyses were computed using MATLAB (The MathWorks, Natick, MA).

### Results

#### *δG* model fit

**Fig. 5:**
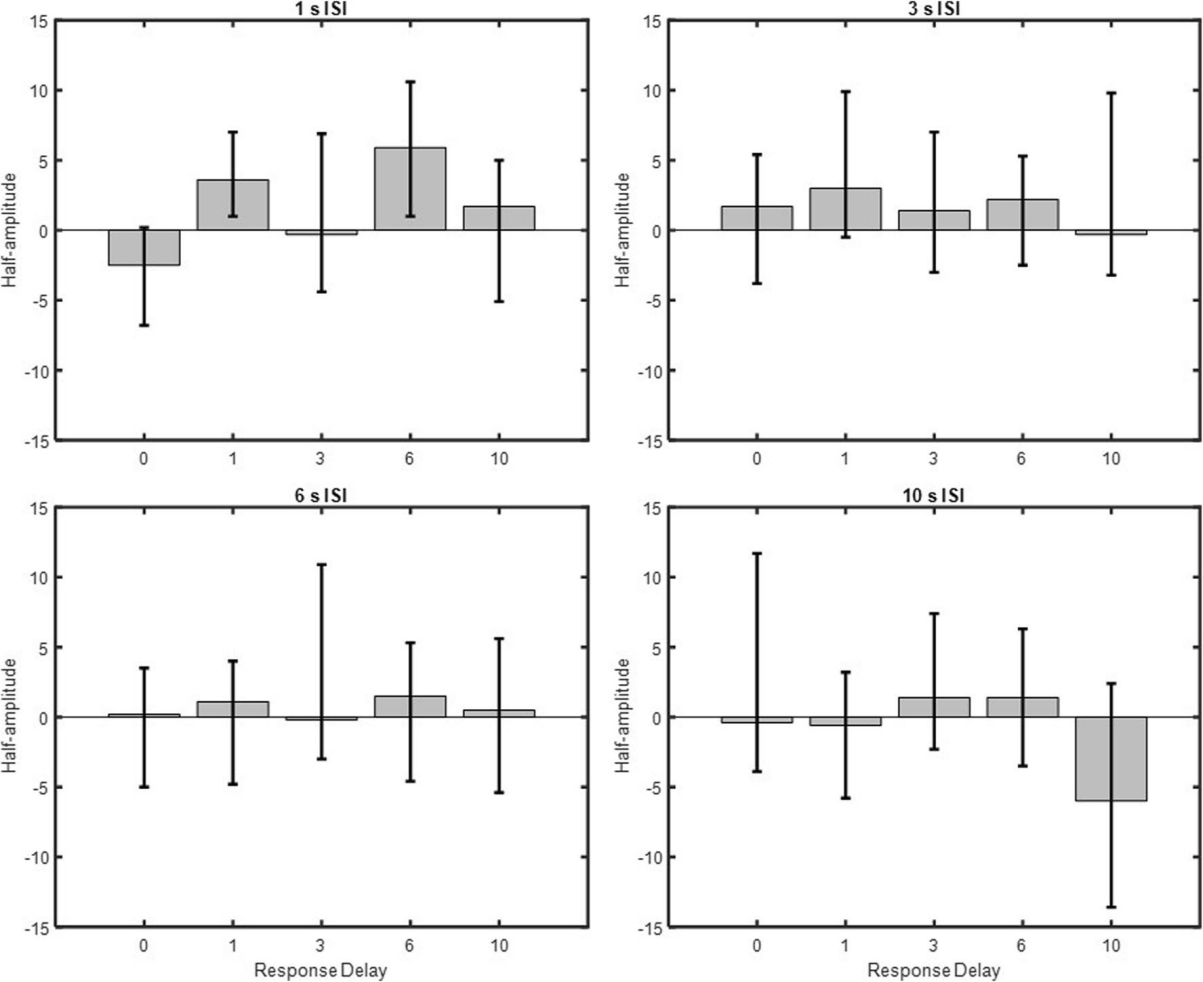
Serial dependence magnitude for all ISI and response delay conditions. Serial dependence for all ISIs and response delays. Half-amplitudes are displayed on the ordinate and response delays in seconds on the abscissa. Error bars represent 95% confidence intervals

As shown in Fig. 5, statistically significant SD was obtained when the ISI was 1 s and the response delay 1 or 6 s (1 s response delay, *a* = 3.6, 95% CI [1, 7], 6 s response delay, *a* = 5.9, 95% CI [1, 10.6], where *a* is the half-amplitude of the *δG* model fit). No statistically significant SD was obtained when the ISI was 1 s and the response delay was 0 s,* a* = −2.5, 95% CI [−6.8, 0.2], 3 s, *a* = −0.3, 95% CI [−4.4, 6.9], or 10 s, *a* = 1.7, 95% CI [−5.1, 5]. Likewise, no statistically significant SD was obtained for the 3, 6, and 10 s ISIs, regardless of response delay, all CIs crossed zero.

In similar vein, no statistically significant SD from the previous trial’s stimuli (trial *n* − 1, Face 1, or Face 2) or the previous trial’s response was obtained for any of the ISI and response delay conditions, all CIs crossed zero.

#### Nonparametric results

**Fig. 6:**
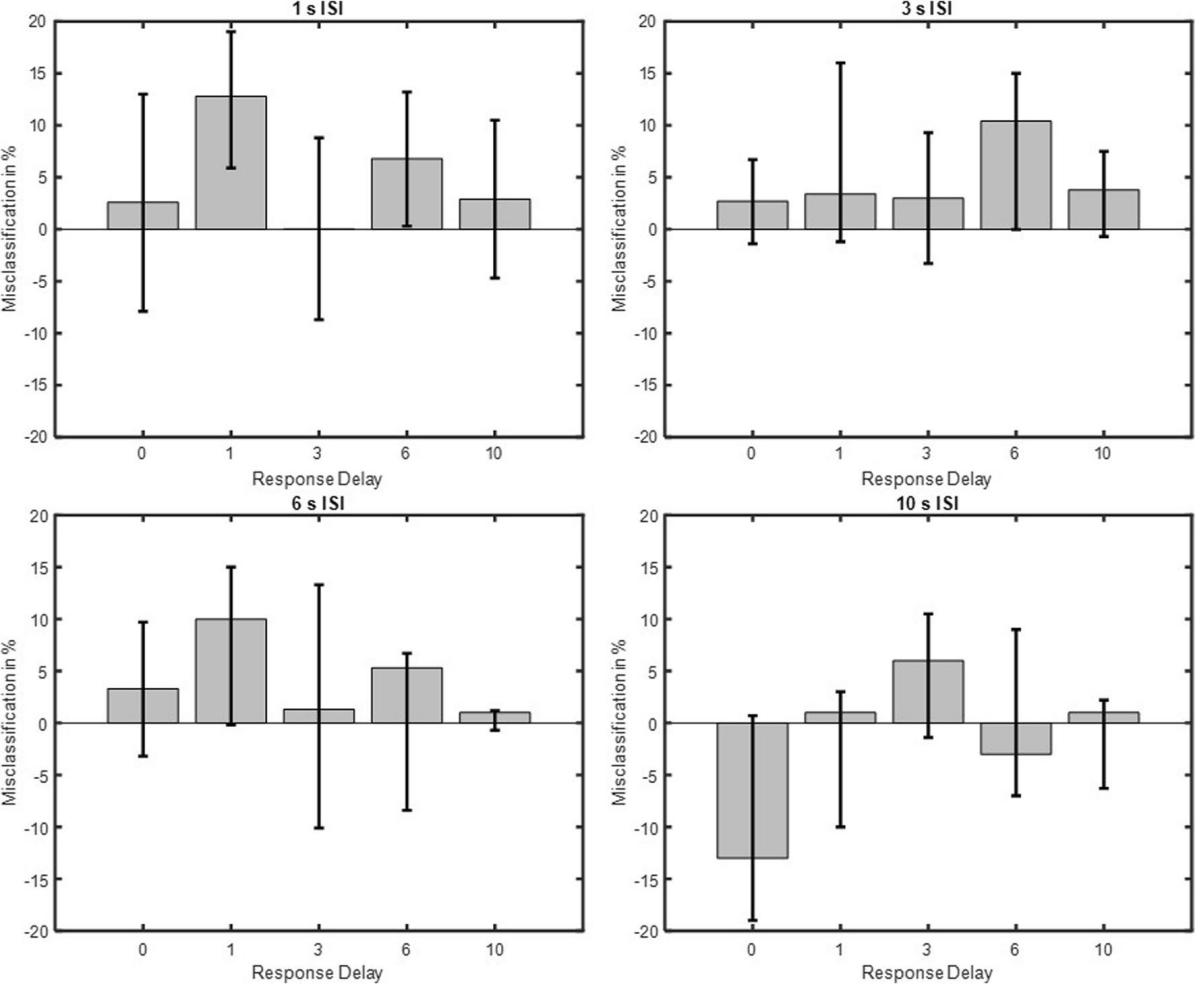
Classification errors for all ISI and response delay conditions. Categorical classification errors for all ISIs and response delays. Classification errors in percent are displayed on the ordinate and response delay in seconds on the abscissa. Error bars represent 95% confidence intervals

The nonparametric analysis confirmed the results from the *δG* model fit. As shown in Fig. 6, statistically significant SD was obtained when the ISI was 1 s and the response delay was 1 or 6 s (1 s response delay = 12.8%, 95% CI [5.9, 19], 6 s response delay = 6.8%, 95% CI [0.3, 13.2]). The nonparametric analysis shows that participants classified Face 2 as belonging to the category of Face 1 12.8% (1-s response delay) and 6.8% (6-s response delay) more often than other face categories. No statistically significant SD was obtained when the ISI was 1 s and the response delay was 0, 3 or 10 s where classification errors were: 2.6%, 95% CI [−7.9, 13], 0.03 %, 95% CI [−8.7, 8.8], and 2.9%, 95% CI [−4.7, 10.5], respectively. Classification accuracy for all 1-s ISI and response delay conditions were: 0-s response delay (73.1±8%), 1-s response delay (72.2±2.3%), 3-s response delay (73.4±5.7%), 6-s response delay (69.4±5.3%), 10 s response delay (69.6±3.2%). No statistically significant SD was obtained for the 3, 6 and 10 s ISIs regardless of response delay, all CIs crossed zero.

### Discussion

Experiment 1 showed that judgments about the identity of faces were attracted toward the previous identity even though participants were explicitly informed that the first face on each trial was irrelevant to the task. Contrary to the assumption that SD is driven by processes related to task relevance (Bae & Luck, 2020; Czoschke et al., 2019; Kang & Choi, 2015; Pascucci et al., 2019; Pascucci & Plomp, 2021), SD was obtained only within a trial, whereas no influence of stimuli from the previous trial was observed. This suggests an effect that did not occur as a result of response or decisional bias in relation to the previously seen face.

SD was found only for short ISI of 1 s, whereas negligible effects were found for ISI of 3 s or longer. An interaction between ISI and response delay also showed that SD occurred at response delays of 1 and 6 s, possibly reflecting early (Cicchini et al., 2017; Manassi et al., 2018) and late stages of visual processing (Bliss et al., 2017; Fritsche et al., 2017). In sum, the results suggest that SD facial identity effects can occur independently of task-related processes but as a result of perceptual and VWM processes.

## Experiment 2

Experiment 1 showed that mere attention to a previous face is a sufficient determinant for SD facial identity effects. SD was found at 1 and 6 s response delays, indicating the involvement of perceptual and postperceptual VWM processes. Sequential effects can occur as a result of interactions between objects within VWM (Bae & Luck, 2017), and distinguishing such effects from SD effects is therefore important for understanding the mechanisms underlying SD.

SD implies “any linear or nonlinear relationship that can be used to predict future events from the past” (Pascucci et al., 2023). Therefore, any influence from a subsequently presented face on judgments of a previous face would not be classified as SD. However, if SD and VWM interactions rely on the same underlying mechanism, a subsequently viewed face should influence the judgement of a previous face similarly to how a previous face influences the judgement of a subsequent face. Likewise, similar temporal dynamics would be expected. Building on the work of Bae and Luck (2017), numerical postcues were used in Experiment 2 to manipulate stimulus priority, so the adjustment response was made to Face 1 on 50% of all trials. Any influence from Face 2 on adjustment responses to Face 1 would likely reflect memory interactions, as Face 1 was already perceived and actively maintained in VWM when Face 2 was presented. The use of postcues also fulfilled the goal of investigating whether the temporal pattern obtained in Experiment 1 would be repeated if both faces had to be actively maintained during the response delay.

### Method

#### Participants

Twenty-four participants took part in Experiment 2 (12 women and 12 men; age range: 19–43 years). Participants were recruited amongst psychology students at Lund University and via the Swedish online recruitment website Accindi (www.accindi.se). All participants reported normal or corrected-to-normal visual acuity, and all received a gift voucher of 100 SEK upon completion of each experimental session. All relevant ethical guidelines were followed, as detailed in Experiment 1.

#### Procedure

**Fig. 7:**
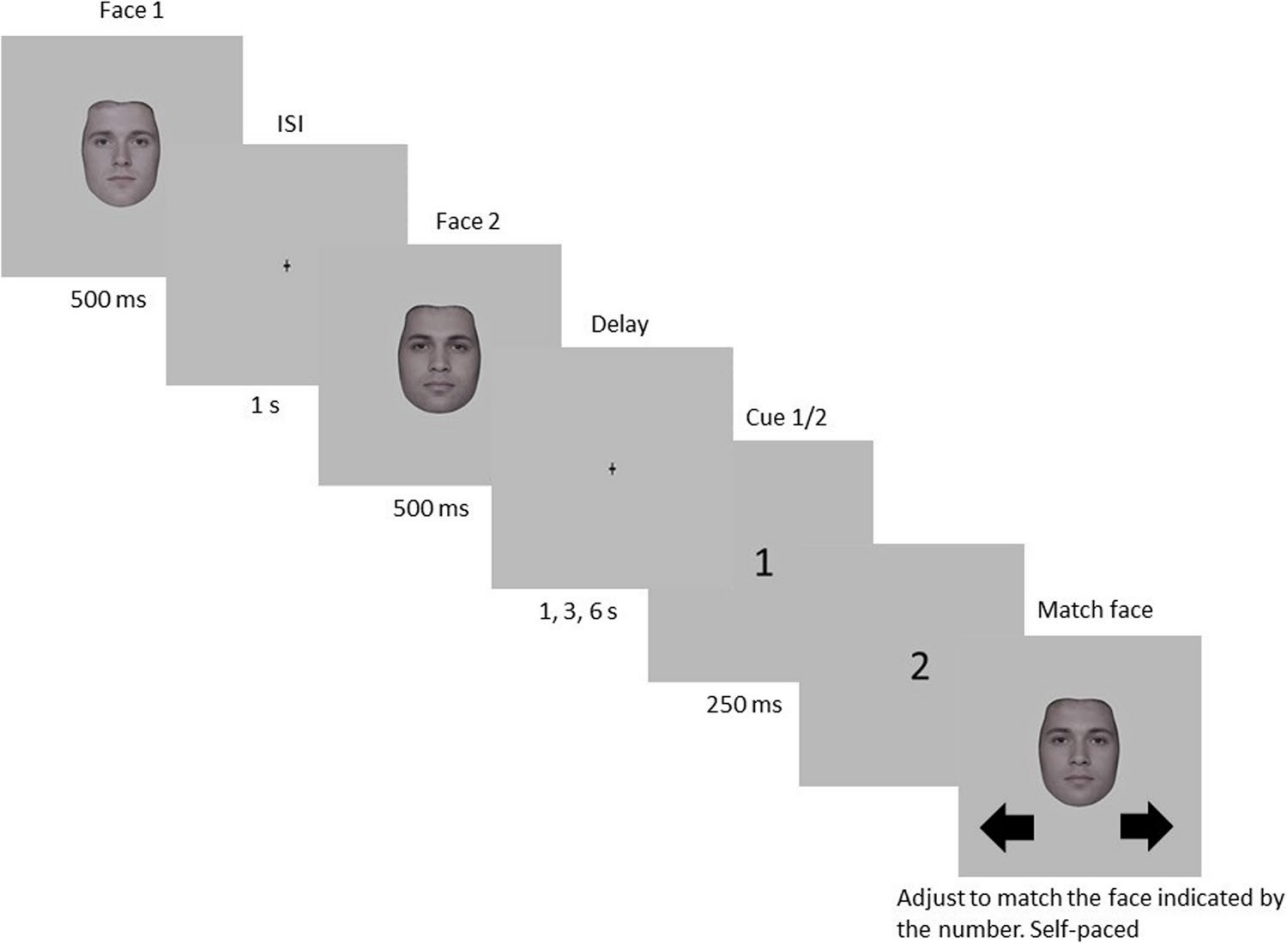
One trial sequence in Experiment 2. Trial sequence in Experiment 2. Each trial began with two randomly presented face images (Face 1 and Face 2), each presented for 500 ms and separated by a 1-s ISI. A fixation cross was present throughout the ISI. After Face 2, a fixation cross was present during a pseudorandom response delay of 1, 3, or 6 s. After the delay and before the response, a postcue (1 or 2) was presented for 250 ms, indicating which face was the target of the current trial. Participants then saw a test screen containing a random adjustment face drawn from the morphed continuum that they matched to the target face indicated by the postcue using the left and right arrow keys (if 1—match to Face 1, if 2—match to Face 2). To register their response, they pressed the down arrow key

The procedure in Experiment 2 was the same as in Experiment 1, except for the following changes: ISIs of 3, 6, and 10 s and response delays of 0 and 10 s were excluded based on the absence of statistically significant effects in Experiment 1. Here, the ISI between Face 1 and Face 2 was held constant at 1 s. Response delays of 1, 3, and 6 s were pseudorandomly selected so each delay was presented an equal number of times during the experimental session. Following the response delay, a numerical postcue was presented for 250 ms to indicate what face was the current trial’s target stimulus. If the postcue was “1,” the adjustment face should be matched to Face 1, and if the postcue was “2,” the adjustment face should be matched to Face 2 (Fig. 7). The postcues were pseudorandomly selected to be presented an equal number of times during the experimental session. Each experimental session comprised 300 trials and took on average 60 minutes to complete. Each participant completed between 300 and 900 trials across one to three separate experimental sessions, totaling 14,205 trials across all participants.

The same computational software as in Experiment 1 was used to run Experiment 2, but now on a Dell Optiplex 5000 MFF computer (Dell Inc., Round Rock, TX). The stimuli were presented on a 24-inch Fujitsu monitor (Fujitsu, Minato, Tokyo) against a light-gray background with a pixel resolution of 1,680 × 1,050 and a refresh rate of 60 Hz.

#### Data analyses

Trials were aggregated into six conditions grouped by response delay (1, 3, 6 s) and target stimulus (Face 1, or Face 2). SD magnitude was computed by fitting a *δG* function to the data following the same procedure as in Experiment 1 across all participants by each target stimulus and response delay condition. The model parameters were the same as in Experiment 1 except for conditions of target stimulus Face 1 where the *y* parameter was set to represent the difference in morph steps between Face 1 and the response. To test for statistically significant half-amplitudes, 95% CIs for each condition were computed using the same resampling procedure described in Experiment 1. A nonparametric analysis was also computed for each condition following the procedure described in Experiment 1. As in Experiment 1, trials with response times >15 s and response errors exceeding±60 morph steps were excluded from the analysis, resulting in the removal of 2.1% of trials.

SD from the previous trial’s stimuli was controlled for using the same procedure as in Experiment 1 by setting the *x* parameter in the model to represent the difference in morph steps between the current trial’s target stimulus and the previous trial’s stimuli or response. Averaged across all participants and conditions mean adjustment time was 4.9 s with a standard deviation of 2.7 s. No statistically significant differences were found between mean adjustment times for the different experimental conditions, all *p*s > .05. All statistical analyses were computed using MATLAB (The MathWorks, Natick, MA).

### Results

#### *δG* model fit

**Fig. 8:**
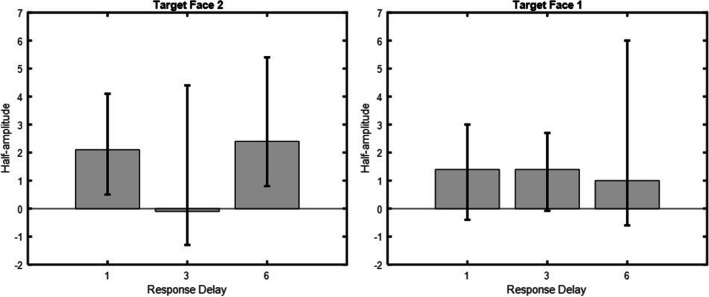
Serial dependence for all target stimulus and response delay conditions. Serial dependence for all conditions. Half-amplitudes are displayed on the ordinate and response delays in seconds on the abscissa. Error bars represent 95% confidence intervals. Target stimulus Face 2 to the left and target stimulus Face 1 to the right in the figure

When the target stimulus was Face 2, statistically significant SD was obtained for the 1 and 6 s response delays, *a* = 2.1, 95% CI [0.5, 4.1], and *a* = 2.4, 95% CI [0.8, 5.4] respectively. As shown in Fig. 8, no statistically significant SD was obtained for the 3 s response delay, *a* = −0.1, 95% CI [−1.3, 4.4]. When the target stimulus was Face 1, no statistically significant SD was obtained for any of the response delays, 1 s response delay (*a* = 1.4, 95% CI [−0.4, 3]), 3-s response delay (*a* = 1.4, 95% CI [−0.08, 2.7], 6-s response delay (*a* = 1, 95% CI [−0.6, 6]). No statistically significant SD was obtained from the previous trial’s stimuli or the previous trial’s response in any of the target stimulus and response delay conditions, all CIs crossed zero.

#### Nonparametric results

**Fig. 9:**
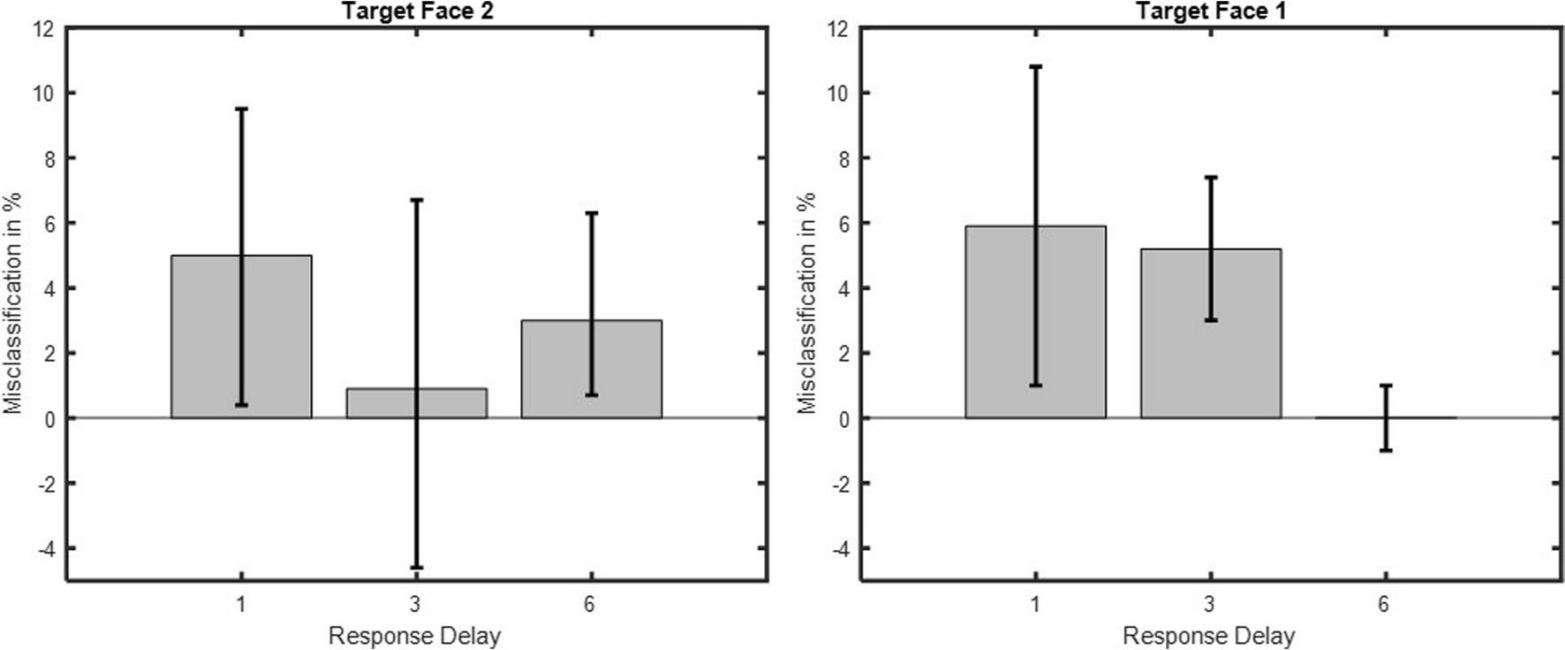
Categorical classification errors for all target stimulus and response delay conditions. Categorical classification errors for all target stimulus and response delay conditions. Categorical classification errors in percent are shown on the ordinate and response delay in seconds on the abscissa. Error bars represent 95% confidence intervals. Target stimulus Face 2 is to the right, and target stimulus Face 1 to the left in the figure

The nonparametric analysis confirmed the results of the *δG* fit when the target stimulus was Face 2. As shown in Fig. 9, participants classified Face 2 as belonging to the category of Face 1 5% (1-s response delay, 95% CI [0.4, 9.5]), 0.9 % (3-s response delay, 95% CI [−4.6, 6.7]), and 3 % (6-s response delay, 95% CI [0.7, 6.3]) more often than other face categories. Classification accuracies when the target stimulus was Face 2 were; 1-s response delay (73.9±2.5%), 3-s response delay (72.2±4%), 6-s response delay (72.6±3.3%), respectively.

The nonparametric analysis did not confirm the results of the *δG* fit when the target stimulus was Face 1. Here, categorical classification errors were statistically significant for the 1 and 3-s response delays. Participants classified Face 1 as belonging to the category of Face 2 5.9% (1-s response delay, 95% CI [1, 10.8]), 5.2% (3-s response delay, 95% CI [3, 7.4]), and 0.01% (6-s response delay, 95% CI [−1, 1]) more often than other face categories. Classification accuracies when the target stimulus was Face 1 were: 1-s response delay (71.2±3.8%), 3-s response delay (71.4±3.8%), 6-s response delay (71.1±4.1%), respectively.

### Discussion

Experiment 2 showed that actively maintaining a previous face during a response delay had a similar effect on identity judgments of a subsequent face as when the previous face was merely attended to. Moreover, the temporal pattern was similar to that obtained in Experiment 1. SD again occurred consistently at response delays of 1 and 6 s, whereas no SD was found at a response delay of 3 s.

Inconsistent results were obtained when the target stimulus was Face 1. Nonparametric analysis revealed significant attractive effects for response delays of 1 and 3 s, whereas *δG* fitting revealed no significant effects for any of the response delays. In addition, the temporal pattern differed from that of target stimulus Face 2 with a decrease in attraction from 1 to 6 s. Given that Face 1 was already perceived when Face 2 was presented suggests that the attractive effects resulted from VWM interactions. Overall, Experiment 2 suggests that SD effects and serial effects arising from VWM interactions rely on separable underlying mechanisms and exhibit different temporal dynamics.

## General discussion

The primary aim of the present study was to investigate the processing stages of SD facial identity effects. More specifically, to investigate whether mere attention to a task-irrelevant face is sufficient for SD to manifest, and the temporal dynamics of such effects. Experiment 1 showed that SD facial identity effects are time-dependent, consistent with expectations of visual continuity within short temporal delays (Burr & Cicchini, 2014). Both Experiment 1 and Experiment 2 provide evidence for a distinct temporal pattern in SD facial identity effects regardless of whether a previous face is merely attended to or actively maintained during a temporal delay. The postcues in Experiment 2 made it possible to isolate SD effects from VWM interactions and to show that they are indeed separate processes that exhibit different temporal dynamics. Taken together, the present results uncover the temporal dynamics of SD facial identity effects and provide evidence for two distinct serial effects that may influence facial identity judgments.

Experiment 1 provides evidence that encoding a previous face is a sufficient determinant for SD. That SD was obtained from a stimulus with no relevance to the current or previous trial’s task contradicts the assumption that the effect depends on task relevance (Bae & Luck, 2020; Czoschke et al., 2019; Kang & Choi, 2015; Pascucci et al., 2019; Pascucci & Plomp, 2021). In addition to task relevance, a stimulus is processed through its emotional valence, which determines its relevance and influences how efficiently the stimulus is processed (Compton, 2003; Kauschke et al., 2019). Faces are emotionally significant stimuli, regardless of expression (Palermo & Rhodes, 2007), so the mere processing of a face may be a sufficient determinant of SD face effects. A study in which an irrelevant distractor face was presented during a retention interval has shown similar results, suggesting that attractive biases may arise from mere attentional processing of an irrelevant distractor face (Mallett et al., 2020). The present results complement those of Mallett et al. (2020) and extend previous findings on the “specialness” of faces as visual stimuli. Moreover, the present results may further suggest that SD results from enhanced perceptual processing rather than task relevance per se.

The results support the notion of SD arising at perceptual (Cicchini et al., 2017; Manassi et al., 2018) and postperceptual stages (Bliss et al., 2017; Fritsche et al., 2017). Whereas Bliss et al. (2017) observed a repulsive effect at a response delay of 0 s for spatial position judgments, reflecting early perceptual iconic memory processes, no reliable repulsive or SD effect was found at a response delay of 0 s in Experiment 1. The duration of iconic memory may depend on stimulus information, and prolonged iconic memory duration may manifest as a result of stimulus complexity (Rensink, 2014). Given the high complexity of faces (Sheehan & Nachman, 2014), a delay of 1 s might still be representative of at least some residual iconic memory traces, which would support the notion of SD arising at an early stage of visual processing. However, that no SD was obtained at a response delay of 0 s could also be due to perceptual interference from the adjustment face (Di Lollo & Dixon, 1988), as no noise masks were used in the present experimental work.

In Experiment 1, participants were explicitly informed that Face 1 was irrelevant to the task to prevent this face from being actively maintained in VWM. However, SD effects obtained at a response delay of 6 s are indicative of VWM processes suggesting that the memory representation of Face 1 was accessible at the time of the response. Faces may indeed be unintentionally stored in VWM even though they are not task relevant (Eitam et al., 2014; Schweinberger et al., 2004). Although implicit memory traces of faces are not very robust (Eitam et al., 2014), such weak memory traces may be sufficient to induce SD. Sequential stimulus presentations can also trigger comparison judgments (Luck, 2008). Indeed, residual memory traces of irrelevant facial identity information have been associated with comparison judgments (Zimmermann & Eimer, 2014). So, it is possible that Face 1 was maintained in VWM and compared with Face 2 at the time of the response. However, some morphed face images were very similar and discrimination between similar faces is more difficult compared with simpler objects (Biederman & Kalocsai, 1997), and may lead to interchange errors and spurious serial effects in VWM tasks (Almeida et al., 2015).

In both experiments, no reliable evidence of repulsion or SD was found at a response delay of 3 s, indicating the characteristics of a temporal window resembling complete independence (Burr & Cicchini, 2014). While most evidence supports a two-component model of visual short-term memory (Luck, 2008), a third component has been proposed between iconic memory and VWM (Sligte et al., 2008). Within this component, which has been reported to persist for approximately 3 seconds, memory representations are fragile and can be easily overwritten (Sligte et al., 2010). So, if SD depends on memory traces of previous stimuli, the effect would disappear when these traces are no longer accessible. Memory traces can also persist in an activity silent manner. Bae (2020) examined the time course of face representations during a 1.5-s response delay and found strong facial identity information at early stages that transitioned to a state of activity silence ~1 s after stimulus offset. However, based on participants’ performance, facial identity information appeared to be accessible at the time of the response, contradicting the results found here and the absence of influence from Face 1 at a 3-s response delay. The capacity of VWM and the precision of memory representations may also vary from trial to trial due to noise generated by neuronal fluctuations (Kinchla & Smyzer, 1967; Raffone & Wolters, 2001). However, the 3-second “dip” was repeated in Experiment 2 and is therefore unlikely to be a purely random effect.

While SD in Experiment 1 was found at short ISI of 1 s, no effects were found at ISI of 3 s or longer. The brain adapts to the temporally correlated statistical structure in natural environments (Huk et al., 2018), and the likelihood of changes in the environment increases with longer compared with shorter temporal delays (Burr & Cicchini, 2014; Cicchini et al., 2018). So, it makes sense that SD was obtained exclusively at short ISI, as temporal correlations within short time lags are naturally stronger and changes are therefore less likely to occur. These results are also consistent with other sequential biases such as repetition priming, where priming effects for unfamiliar faces have been found most consistently within short temporal intervals (Bentin & Moscovitch, 1988). Yet, exactly how the mechanisms underlying these two sequential biases differ remains to be determined.

The temporal pattern observed here differs from that of previous findings of SD between trials, which can extend back as far as ~15 s (Bilacchi et al., 2022; Liberman et al., 2014; McKeown et al., 2020). Given that SD may occur at multiple processing stages (Whitney et al., 2022), between-trial effects make it difficult to isolate perceptual and VWM effects from effects resulting from response and decisional biases. Indeed, it has been suggested that SD between-trial effects result from decision processes (Pascucci et al., 2019). However, previous attempts to distinguish such processes have focused on stimuli with low complexity, whereas here two consecutive facial stimuli were presented on each trial. It is therefore possible that the challenge of two facial stimuli prompted active removal of the stimuli from the previous trial to release VWM capacity needed for the task of the current trial (Eng et al., 2005; Lewis-Peacock et al., 2018; Towler et al., 2016), preventing any interference from the previous trial’s stimuli. This could imply that VWM capacity is required for SD facial identity effects to manifest.

The magnitude of SD appeared weaker in Experiment 2 than in Experiment 1 when Face 2 was the target stimulus, which was particularly evident for the 6-s response delay. Retention of multiple faces during prolonged time delays poses a considerable challenge on capacity limited working memory resources (Eng et al., 2005; Towler et al., 2016), and robustness is affected by attentional shifts between memory items during retention (McKeown et al., 2014). So, the simultaneous storage of both faces in VWM may have prevented SD from taking full effect. Consequently, this may be further evidence that VWM capacity is required for SD to occur.

The results of the nonparametric analysis did not support the results of the *δG-*model fit in Experiment 2 when the target stimulus was Face 1. The nonparametric results showed an attractive bias for response delays of 1 and 3 s, whereas no such effect was found based on the *δG* model-fitting results. A temporal pattern more consistent with memory decay and information loss as a function of time was found when the target was Face 1 (Mercer & Barker, 2020). Given that Face 1 was already perceived when Face 2 was presented rules out perceptual influence and suggests that the attractive bias originated from VWM interactions. SD typically occurs between more similar stimuli (Kiyonaga et al., 2017), whereas VWM interactions have been shown to occur when the stimuli are more different (Bae & Luck, 2017), and such effects are not well captured by the *δG* model (Bliss et al., 2017). It is therefore possible that the attractive bias in Face 1 responses toward Face 2 arose between larger stimulus differences that were not captured by the parametric analysis. The inconsistent results observed for target Face 1 could potentially be caused by a recency effect in which Face 1 was masked, or overwritten to some extent, by Face 2 when both faces were relevant to the task (Hanna & Loftus, 1993; Kool et al., 2014). However, a recency effect would likely have resulted in a strong attractive bias in Face 1 judgments toward Face 2, which was not observed, so it seems unlikely that such a recency effect affected the results. Moreover, the experimental stimuli in the present study were continuous in nature, so a categorical analysis consequently introduces some data loss which could have affected the results.

Taken together, the two experiments reported here show that SD facial identity effects have distinct temporal dynamics and suggest that SD may arise as a result of perceptual and VWM processes independent of task-related processes. However, it remains possible that SD requires VWM capacity to manifest, and future research should investigate the precise nature of what appears to be a perceptual-VWM interaction underlying such effects.

## Data Availability

The datasets generated and analyzed during the current study are available from the corresponding author on reasonable request.

## Code availability

The MATLAB scripts generated during the current study are available from the corresponding author on reasonable request.
